# Neural Effects of Cognitive Behavioral Therapy in Psychiatric Disorders: A Systematic Review and Activation Likelihood Estimation Meta-Analysis

**DOI:** 10.3389/fpsyg.2022.853804

**Published:** 2022-05-03

**Authors:** Shiting Yuan, Huiqin Wu, Yun Wu, Huazhen Xu, Jianping Yu, Yuan Zhong, Ning Zhang, Jinyang Li, Qianwen Xu, Chun Wang

**Affiliations:** ^1^Nanjing Brain Hospital Affiliated to Nanjing Medical University, Nanjing, China; ^2^School of Psychology, Nanjing Normal University, Nanjing, China; ^3^Functional Brain Imaging Institute of Nanjing Medical University, Nanjing, China; ^4^Cognitive Behavioral Therapy Institute of Nanjing Medical University, Nanjing, China

**Keywords:** cognitive behavioral therapy (CBT), psychiatric disorder, neuroimaging, meta-analysis, brain network

## Abstract

**Background:**

Cognitive behavioral therapy (CBT) is a first-line psychotherapeutic treatment that has been recommended for psychiatric disorders. Prior neuroimaging studies have provided preliminary evidence suggesting that CBT can have an impact on the activity of brain regions and functional integration between regions. However, the results are far from conclusive. The present article aimed to detect characteristic changes in brain activation following CBT across psychiatric disorders.

**Method:**

Web of Science, Cochrane Library, Scopus, and PubMed databases were searched to identify whole-brain functional neuroimaging studies of CBT through 4 August 2021. To be included in the meta-analysis, studies were required to examine functional activation changes between pre-and post-CBT. The included studies were then divided into subgroups according to different task paradigms. Then, an activation likelihood estimation algorithm (ALE) was performed in the different meta-analyses to identify whether brain regions showed consistent effects. Finally, brain regions identified from the meta-analysis were categorized into eight functional networks according to the spatial correlation values between independent components and the template.

**Results:**

In total, 13 studies met inclusion criteria. Three different meta-analyses were performed separately for total tasks, emotion tasks, and cognition tasks. In the total task ALE meta-analysis, the left precuneus was found to have decreased activation. For the cognition task ALE meta-analysis, left anterior cingulate (ACC) and left middle frontal gyrus (MFG) were found to have decreased activation following CBT. However, the emotion task ALE meta-analysis did not find any specific brain regions showing consistent effects. A review of included studies revealed default mode network (DMN), executive control network (ECN), and salience network (SN) were the most relevant among the eight functional networks.

**Conclusion:**

The results revealed that the altered activation in the prefrontal cortex and precuneus were key regions related to the effects of CBT. Therefore, CBT may modulate the neural circuitry of emotion regulation. This finding provides recommendations for the rapidly developing literature.

## Introduction

Cognitive-behavioral therapy (CBT) is a first-line psychotherapeutic treatment that has been proven effective in treating a variety of psychiatric disorders such as major depressive disorder (MDD), anxiety disorder (AD), and obsessive-compulsive disorder (OCD) (Butler et al., 2006; Beck and Dozois, 2011; Hofmann et al., 2012; McMain et al., 2015). This psychotherapy, pioneered by Ellis (1962) and Beck (1970), focuses on identifying maladaptive cognitions and modifying behavioral patterns to alleviate clinical symptoms and improve function (Beck and Dozois, 2011; Hofmann et al., 2012). Moreover, CBT may promote change in conflict and inflexible appraisals of emotional, cognitive, physiological, and social states by reducing avoidance in experience and behavior (McMain et al., 2015).

Many studies have demonstrated the efficacy of CBT in psychiatric disorders (Butler et al., 2006; Hofmann et al., 2012; Van Dis et al., 2020; Matsumoto et al., 2021). 269 quantitative meta-analyses were identified in a review to verify the effectiveness of CBT for psychiatric disorders. And a subsample of 11 meta-analyses was compared response rates between CBT and other treatments. The result showed CBT was highly effective for depression, anxiety disorders, cannabis, and nicotine dependence. And CBT demonstrated superior efficacy as compared to other forms of psychotherapies in personality disorders, bulimia, positive symptoms in schizophrenia (Hofmann et al., 2012). A study showed that a pooled effect size (Hedge g) post-treatment of −0.49 (95% CI −0.68 to −0.29), which indicated CBT had a significant effect on psychiatric disorders (Matsumoto et al., 2021). Moreover, much evidence addressed that CBT produced long-term persistence of therapeutic effects following the termination of treatment (Butler et al., 2006; Van Dis et al., 2020). A meta-analysis suggested CBT was related to symptom improvement in social anxiety disorder (Hedges g, 0.42; *k* = 3), generalized anxiety disorder [Hedges g, 0.22; the number of studies (*k*) = 10], posttraumatic stress disorder (Hedges g, 0.84; *k* = 5), after 2-month follow-up (Van Dis et al., 2020). Similar findings were observed in depression. A meta-analysis found that there was the same effect between antidepressant medication and CBT at follow-up (Cuijpers et al., 2013).

With the development of neuroimaging technology, many non-invasive techniques such as magnetic resonance imaging (MRI), diffusion tensor imaging (DTI), functional magnetic resonance imaging (fMRI), single-photon emission computed tomography (SPECT), and positron emission tomography (PET) are being utilized to identify structural and functional brain alterations in psychiatric disorders. Whether the subject was in the resting state or in performing a specific task, changes in blood oxygenation level-dependent can be monitored through fMRI (Chen and Glover, 2015). Thus, task-based fMRI was employed to identify functional neuroanatomical networks associated with specific task states (Baggio and Junqué, 2019).

Previous neuroimaging studies have reported abnormalities in brain regions and networks in psychiatric disorders (Goodkind et al., 2015; McTeague et al., 2020). However, most studies focused on structural and functional brain alterations of a specific diagnosis. Although different psychiatric disorders had characteristic clinical presentations, there have common functional impairments in cognition, emotion, behavior, and socio-occupational impairment. Previous transdiagnostic studies had evidenced that the abnormalities in the insula, the dorsal anterior cingulate (dACC), the dorsolateral prefrontal cortex (dlPFC) were associated with cognitive dysfunction (Goodkind et al., 2015). And hyperconnectivity was identified between the salience network (SN), the default mode network (DMN), and the frontoparietal network (FPN). These network alterations were related to cognitive deficits (Sha et al., 2019). Furthermore, the disrupted emotional processing was associated with abnormal activation in the prefrontal regions, the amygdala, the hippocampal/parahippocampal gyri, the thalamus, and the fusiform gyrus (McTeague et al., 2020).

Neuroimaging of CBT has increased enormously over the past several decades and increasing evidence has already documented the relationship between neural changes and symptomatic improvement following CBT for psychiatric disorders.

There exists fairly robust evidence documenting alterations in prefrontal cortical regions and functionally related structures following CBT. A systematic review showed that the most common regions altered by CBT included ACC, posterior cingulate cortex (PCC), and orbitofrontal cortex/dorsomedial prefrontal cortex (OFC/VLPFC). Furthermore, the decreased activity in dorsal anterior cingulate (dACC) following CBT was in line with a model of information processing that described a ventral affective circuit and a dorsal cognitive circuit (Franklin et al., 2016). Another review identified that CBT was related to bilateral deactivation of ACC for specific phobia (Ipser et al., 2013). In addition, a meta-analysis found that compared with healthy controls, activation of ACC/PFC was significantly decreased in negative valence disorders, though activity was normalized in patients following CBT (La Buissonniere-Ariza et al., 2021). Furthermore, a study identified that activation of PFC and posterior cingulate cortex (PCC) were decreased after CBT in insomnia. This study suggested that PFC and PCC were associated with sleep-related attention (Kim et al., 2017). Perhaps CBT affects cognitive and emotional processes through ACC (La Buissonniere-Ariza et al., 2021).

Similarly, altered activation was also reported in the insula and amygdala. For example, a meta-analysis identified that the insula and amygdala were consistently responsive to phobic stimuli, but insula activity normalized following CBT (Ipser et al., 2013). This suggests that CBT could improve threat-safety discrimination (La Buissonniere-Ariza et al., 2021). In the same line, post-treatment reductions in the insula and amygdala response to emotion perception were related to greater clinical improvement in adults with anxiety and/or depression (Gorka et al., 2019). It appears that reductions in insula and amygdala activity are a sign of successful CBT intervention in MDD (Zhou et al., 2021). In anxiety disorders, deactivation of the amygdala has also been observed following CBT. In other negative valence disorders, greater pre-treatment activation to emotional stimuli predicted greater reductions in clinical symptoms at post-treatment (La Buissonniere-Ariza et al., 2021).

In addition to the aforementioned brain regions, there are other cortical regions (hippocampus, thalamus, precentral and postcentral gyri) that may potentially be associated with treatment response (Ipser et al., 2013; La Buissonniere-Ariza et al., 2021). However, studies involving these regions are inconsistent.

As evidence mounts, it is clear that information related to affective and cognitive processes is integrated between brain regions. CBT responses have been linked to functional connectivity in the default mode, cognitive control, salience, and frontoparietal networks in psychiatric disorders (Mason et al., 2016; La Buissonniere-Ariza et al., 2021). Besides, previous studies have shown that the dysfunctional connectivity in prefrontal-limbic regions led to conflicts in cognitive processes during the experience of negative affect (Mason et al., 2016). Thus, not only does CBT alter the activity of individual brain regions, but it can also have an effect on functional integration between regions.

Despite the increased interest sparked by neuroimaging studies that assessed brain modifications after CBT, most of them focused on specific disorders or populations with common symptom characteristics. Few neuroimaging studies directly explored neural mechanisms underlying CBT across psychiatric disorders. The impact of CBT on the brains of patients with psychiatric disorders is not clear. The present study aimed to use systematic reviews and meta-analyses to identify neural changes associated with CBT that are common across psychiatric disorders. Furthermore, we selected studies that used task paradigms that focused on the relationship between cognition, emotion, and behavior since disrupted emotional processing and cognitive deficits are common features of multiple psychiatric disorders. In the present study, two coordinate-based ALE meta-analyses were conducted to investigate the changes in cognitive or emotion-related brain regions following CBT. The present systematic review and meta-analysis will provide a clearer view of the current state of research and will identify areas for further investigation.

## Methods

### Search and Inclusion of Studies

A comprehensive and systematic search was conducted using the Pubmed, Cochrane Library, Scopus, and Web of Science electronic databases before 4 August 2021. This search was based on the following keywords and combinations: (“cognitive behavior* OR cognitive therapy OR behavior therapy OR CBT”) AND (“MRI or magnetic resonance imaging or fMRI or functional magnetic resonance imaging or PET or positron emission tomography or SPECT or single photon emission computed/tomography”).

**FIGURE 1:**
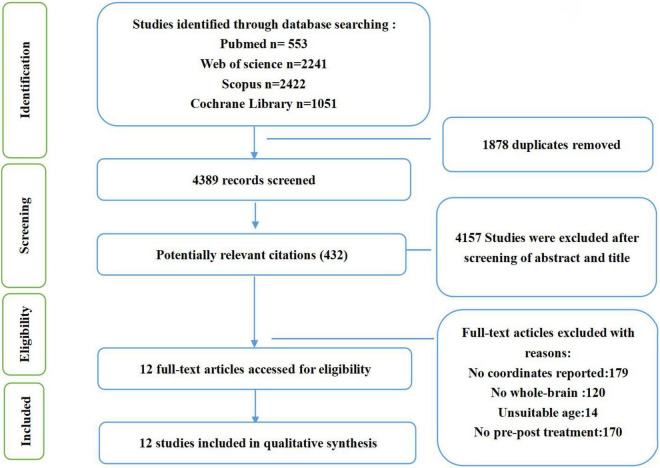
Flowchart of the searching strategy and study selection process, based on PRISMA template (Liberati et al., 2009; Moher et al., 2009).

Inclusion criteria required studies to (1) be part of a clinical trial examining cognitive behavioral therapy in psychiatric disorders; (2) compare the neural changes between pre-treatment and post-treatment using fMRI during a task; (3) conduct whole-brain voxel-wise analyses; (4) report results as coordinates in standard Talairach or MNI space; (4) publish in English. Some studies were excluded due to: (1) presenting only baseline coordinates; (2) reporting resting-state fMRI, functional connectivity, voxel-based morphometry, or region-of-interest analyses. If different studies or tasks used the same subjects, we included the study or task which had the largest sample. And Studies that found no significant differences were excluded. The entire search process is shown in the flowchart below (Figure 1).

### Activation Likelihood Estimation Methodology

For the resulting articles, all eligible experiments were entered into the BrainMap database according to paradigm and direction of effect. Eligible coordinates were entered into the database in MNI (Montreal Neuroimaging Institute) space. Coordinates presented in Talairach space were transformed to MNI space using GingerALE. The meta-analysis was performed in GingerALE 3.0.2 software downloaded from the BrainMap website to examine whether there was a significant overlap across multiple studies. The ALE algorithm converted the reported foci into spatial centers of 3-dimensional Gaussian probability distributions to model a modeled activation (MA) map (Eickhoff et al., 2009, 2012). The union of activation probabilities for each focus in the MA map was calculated to obtain ALE scores. And the included studies had been weighted by ALE algorithms. ALE algorithms weighted the localizing power of individual studies by building the proposed uncertainty model.

The 13 studies identified in the literature search included emotion or cognition task paradigms. According to the differences of paradigms, three coordinate-based ALE meta-analyses were separately performed to determine consistency across neuroimaging studies. The ALE meta-analyses were performed on brain areas displaying altered activation with *P*-value = 0.05, cluster-level = 0.05 and threshold permutations = 1,000.

Then, brain regions displaying alterations were categorized into eight functional networks according to the spatial correlation values between independent components and the template. These eight brain networks were defined mainly according to Shirer’s definition of 90 ROI functions (Shirer et al., 2012).

## Results

### Studies Included

According to searching procedures, thirteen studies were assessed for eligibility. In these retained studies, 9 used paradigms classified as emotion, and 4 used paradigms classified as cognition, as shown in Table 1. The 13 articles included 1 study for patients with psychosis, 1 for the cocaine-dependent disorder, 1 for eating disorder, 4 for major depressive disorder, and 6 for anxiety disorder. Patients in these studies were aged from 11 to 60, including patient groups and control groups.

**TABLE 1:** Characteristics of 13 included studies.

Author	Disorder	Patients/n	Control patients/n	Healthy/n	Mean age	Gender (male/female)	Task		Sessions	Contrasts
							Cognition	Emotion		
Rubin-Falcone et al., 2020	MDD	32	–	19	34.8	15/16	–	Emotional reactivity and emotion regulation	14 sessions over 12 weeks	Pre > Post
Hauner et al., 2012	SP	12	–	–	22.3	3/9	–	Emotion photogenic image	2 h	Pre < Post Pre > Post
Fonzo et al., 2014	GAD	21	–	11	34.29	16/5	–	Facial emotion processing	10 weeks	Pre > Post
Fu et al., 2008	MDD	16	–	16	40	6/2	–	Affect recognition	16 weeks	Pre < Post Pre > Post
Sankar et al., 2015	MDD	16	–	16	39.9	13/3	–	Dysfunctional attitudes	16 weeks	Pre > Post
Månsson et al., 2013	SAD	13	13	–	32.46	2/11	–	Affective face processing	12 weeks	Pre < Post Pre > Post
Reinecke et al., 2018	AD	14	14	–	34.8	5/9	–	Emotion regulation	4 weeks	Pre > Post
Kumari et al., 2011	Psychosis	28	28	–	35.68	9/11	–	affect processing	6–8 months	Pre > Post
Kircher et al., 2013	PD	21	21	42	35.42	29/42	–	Fear conditioning	12 weeks	Pre > Post
Yoshimura et al., 2014	MDD	23		15	30.5	6/2	Self-referential	–	12 weeks	Pre > Post
Devito et al., 2012	SUD	12	–	12	37.2	7/5	Stroop	–	8 weeks	Pre > Post
Vocks et al., 2011	ED	32	–	17	26.12	9/8	Body image	–	10 weeks	Pre > Post
Bomyea et al., 2020	AD	30	–	–	36.6	11/15	Reappraisal-based emotion regulation	–	10 sessions over 12 weeks	Pre > Post

### Activation Likelihood Estimation Analysis

#### Activation Likelihood Estimation Meta-Analysis of Cognition Task Following Cognitive Behavioral Therapy

**FIGURE 2:**
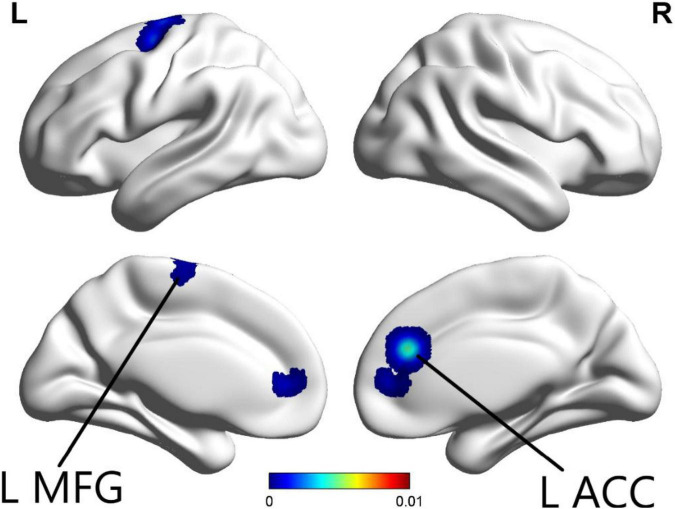
In the cognition paradigm, significantly decreased activations across the left anterior cingulate (L ACC) and left middle frontal gyrus (L MFG) were found in patients with psychiatric disorders after CBT (*p* < 0.05).

The ALE meta-analysis was performed on brain areas displaying altered activation during cognition tasks from pre to post-CBT with *P*-value = 0.05, cluster-level = 0.05 and threshold permutations = 1,000. The results showed that the left anterior cingulate (L ACC) and left middle frontal gyrus (L MFG) demonstrated significantly decreased activation (Figure 2 and Table 2).

**TABLE 2:** Activation areas resulting from meta-analysis, peak voxels, ALE values, and cluster sizes are included.

Cluster size (mm^3^)	ALE	MNI-coordinates			Brain region
		X	V	z	
**Cognition task**					
1,549	0.008694564	0	46	6	Left anterior cingulate
1,066	0.005506158	−18	−12	61	Left middle frontal gyrus
**All task**					
1,393	0.009742522	−16	−72	12	Left cuneus

#### Activation Likelihood Estimation Meta-Analysis of Emotion Task Following Cognitive Behavioral Therapy

For emotion tasks, we performed the same procedure. No clusters showed significant overlap in ALE maps.

#### Activation Likelihood Estimation Meta-Analysis of All Tasks Following Cognitive Behavioral Therapy

**FIGURE 3:**
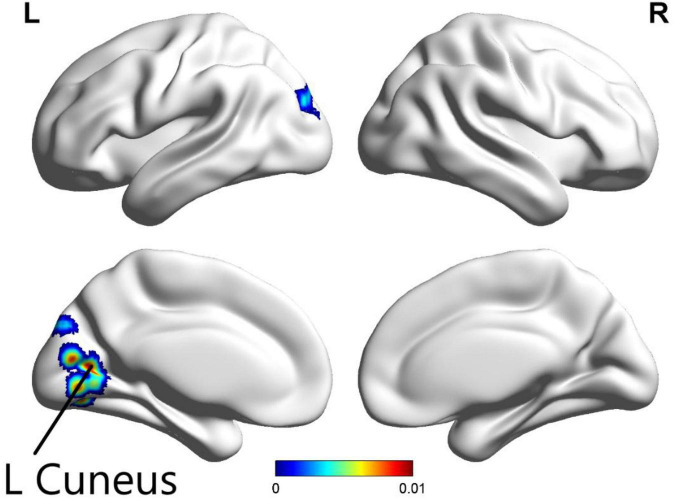
In all paradigms, significantly decreased activation in the left cuneus was found in patients with psychiatric disorders after CBT (*p* < 0.05).

The ALE meta-analysis was performed on brain areas displaying altered activation during all tasks from pre to post-CBT with *P*-value = 0.05, cluster-level = 0.05 and threshold permutations = 1,000. The results revealed that the left cuneus displayed significantly decreased activation (Figure 3 and Table 2).

### Brain Functional Alterations and Related Brain Networks Following Cognitive Behavioral Therapy

**FIGURE 4:**
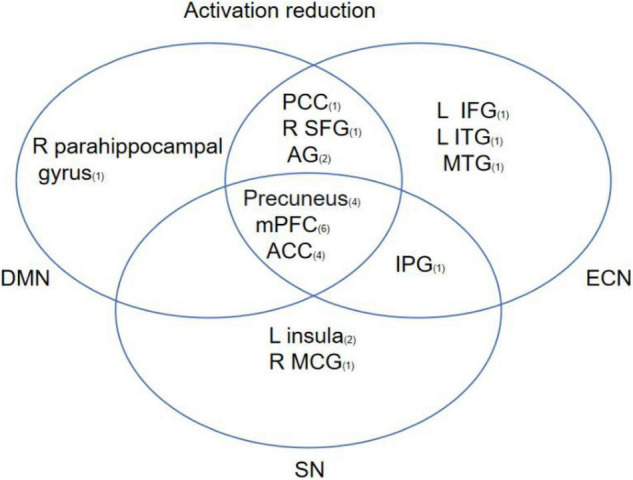
This figure shows regions that have been reported to show CBT-related changes and the overlap between regions involved in different brain networks. The subscript number next to the brain region represents the number of articles related to that brain region.

Based on the 13 studies reviewed, the most consistent findings were altered activation between the prefrontal cortex and other limbic regions following CBT. To better understand the effects of CBT on psychiatric patients, brain regions from included studies were categorized into eight functional networks according to the spatial correlation values between independent components and the template. The results showed that 9 brain areas reported reduced activation in DMN including the medial prefrontal cortex (mPFC), ACC, precuneus, PCC, left angular gyrus (AG), left middle occipital gyrus (MOG), right superior frontal gyrus (SFG), right parahippocampal gyrus, and right angular gyrus. 11 brain areas reported reduced activation in ECN including mPFC, ACC, PCC, L AG, precuneus, R SFG, L SPG, L IPG, left inferior temporal gyrus (L ITG), and middle temporal gyrus (MTG). 6 brain areas reported reduced activation in SN including mPFC, precuneus, left insula, ACC, IPG, and right midcingulate gyrus (MGC). The most common outcome across studies was decreased activation in mPFC, ACC, and precuneus (Figure 4).

## Discussion

In the present study, a systematic review and meta-analysis were conducted to identify brain regions and related brain networks associated with the effects of CBT in patients with psychiatric disorders. And to our knowledge, this review is the first to discover altered brain activation associated with CBT in task-fMRI. First, the most commonly reported finding was decreased activation in mPFC, ACC, and precuneus from pre to post-treatment. After reviewing all included studies, connectivity within DMN, ECN, and SN was connected with the effects of CBT. Second, the ALE meta-analysis of cognition fMRI studies revealed that the left ACC and left MFG showed significant overlap across studies. Third, the ALE meta-analysis of all fMRI studies revealed that left precuneus showed significant overlap across studies.

For the ALE-meta-analysis of cognition tasks, significantly decreased activation across left ACC and left MFG was found. After referencing previous studies and reviewing the literature, this was an expected finding following CBT. The mPFC is a region that consists ACC, MFG, prelimbic cortex (PL), infralimbic cortex, and medial precentral area. It plays a critical role in emotion regulation, cognitive processing, motivation, and social interaction (Riga et al., 2014; Xu et al., 2019). The dysfunction of mPFC found in psychiatric disorders is related to loss of social skills, motivation deficiency, and dysregulation of cognition and emotion (Xu et al., 2019). Moreover, ACC, as one of the core regions of mPFC, is also involved in emotional processing, higher cognition, motivation, and motor control (Monosov et al., 2020). There was a multitude of evidence in functional imaging studies reporting altered activation of mPFC/ACC following CBT. In one study, from pre- to post-treatment, decreased activation in ACC during fear/angry faces was observed in generalized anxiety disorder (Fonzo et al., 2014). In another study, compared with control groups, depressive patients displayed increased activation in the MPFC during the self/negative condition. In a third study, MPFC and ventral anterior cingulate cortex (vACC) displayed attenuated activation during self-referential processing of negatively valenced words after 12 sessions of CBT (Yoshimura et al., 2014). A recent review examined the involvement of ACC and mPFC in appraisal and expression of negative emotion and found that ACC/mPFC exhibited a modulating effect on limbic regions involved in producing emotional responses (Etkin et al., 2011). The evidence suggests that CBT may participate in emotional processing by acting on mPFC and ACC (Fonzo et al., 2014; Yoshimura et al., 2014).

In the present systematic review and meta-analysis, we also found altered activation of the precuneus. According to the ALE meta-analysis results for all tasks, significantly reduced activation was found in the left cuneus. The precuneus and mPFC are identified as core parts of DMN, which is implicated in various psychiatric disorders (Whitfield-Gabrieli and Ford, 2012). Previous studies have shown that the precuneus performs an important role in a wide range of higher-order cognitive functions including visuospatial imagery, episodic memory retrieval, self-processing, and consciousness (Cavanna and Trimble, 2006). One study reported that greater BOLD deactivation during emotion regulation from pre- to post-treatment in the precuneus was associated with better treatment outcomes (Rubin-Falcone et al., 2020). Similarly, post-treatment increases in precuneus activity were also observed in specific phobia, suggesting that CBT may effectively improve threat-safety discrimination. The abnormal increases in this parietal region suggest a compensatory mechanism of cognitive function. It may be that the precuneus reorganizes the process of emotion stimuli (Álvarez-Pérez et al., 2021). On the other hand, a previous study has shown that self-referential processing is regulated by emotion (Qian et al., 2020). Given the role of the precuneus in self-referential processing, this may reflect greater disengagement in self-referential processing as a function of successful CBT (Herold et al., 2016). Furthermore, effects of CBT were reported across several other brain regions including parietal, occipital, temporal, and limbic regions, suggesting that many brain regions are important for cognition and emotion and may be sensitive to the effects of CBT. However, studies involving these regions are inconsistent.

In the present study, DMN, ECN, and SN were the most relevant networks after reviewing eligible neuroimaging data. The DMN is involved in emotional processing, self-referential processing, and the recollection of prior experiences (Whitfield-Gabrieli and Ford, 2012; Mohan et al., 2016; Zhang and Volkow, 2019; Yeshurun et al., 2021). DMN exhibits hyperactivity during resting states and deactivation during the performance of cognitive tasks when attention is directed externally (Broyd et al., 2009; Whitfield-Gabrieli and Ford, 2012; Andrews-Hanna et al., 2014; Simon and Engström, 2015). Additionally, previous studies showed that abnormal intra-network connectivity of the DMN was associated with abnormalities in cognitive function including self-referential and introspective mental activity, attention, and working memory across psychiatric disorders (Broyd et al., 2009; Vuper et al., 2021). From pre- to post-CBT, normalization of intra-functional connectivity in DMN was positively correlated with clinical improvement in OCD. The evidence suggests that CBT affects the intra-functional connectivity in DMN to modulate cognitive function. In addition, connectivity changes in ECN and SN have also been observed following CBT. The ECN is engaged in a broad spectrum of high-level cognitive functions including planning, decision making, attention regulation, and working memory (Menon, 2011; Xu et al., 2020). SN is associated with regulating emotional and sensory stimuli, adjusting cognitive directivity, and allocating attention (Seeley, 2019). Previous studies have shown that the connectivity between the DMN, the SN, and the CEN impacts cognitive functions and dysfunction of intrinsic wiring, and connectivity in the three core neurocognitive networks has been evidenced in psychiatric disorders (Menon, 2011; Vuper et al., 2021). For instance, in post-traumatic stress disorder (PTSD), altered DMN and SN connectivity was related to the reduction of clinical symptoms following CBT (La Buissonniere-Ariza et al., 2021; Vuper et al., 2021). This result suggests that CBT improves cognitive function by affecting the internal connection of the brain network and the interaction among brain networks. In the present meta-analysis, we chose to include all available studies due to the lack of available CBT fMRI studies in the literature. Although emotion-task ALE meta-analysis did not find any specific brain regions showing consistent effects, the results indicated that CBT had broad rather than specific effects on task-evoked BOLD response across the brain.

### Limitations

The present study has several limitations. First, the number of studies in the present ALE meta-analysis was small. It limited our ability to detect more accurate ALE results. To meet the challenges of research heterogeneity and ALE methods, a comprehensive literature search with relatively strict inclusion criteria and strict correction procedures was used to improve the internal effectiveness of the study, which is consistent with previous ale studies. Second, due to the scant amount of literature and uneven types of diseases, our data is not representative of psychiatric disorders as a whole. Third, confounding factors, such as age and gender, are unavoidable limitations.

## Conclusion

The present systematic review and ALE meta-analysis was the first summary of the available literature on core neural regions that are related to CBT in task-fMRI. Findings from the present study indicated that CBT was associated with significantly decreased activity in mPFC/ACC and precuneus. Significant changes in neural activity were also identified in intrinsic wiring and connectivity in DMN, ECN, and SN, suggesting that these effects may mediate cognitive improvements and emotion regulation.

## Data Availability Statement

The original contributions presented in the study are included in the article/supplementary material, further inquiries can be directed to the corresponding author/s.

## Author Contributions

CW, SY, HW, NZ, and YW contributed to initiating, designing the study, and collecting the data. SY, QX, JY, HX, and JL made substantial contributions in writing the manuscript. YZ provided editing and writing assistance. All authors reviewed and approved the final manuscript.

## Conflict of Interest

The authors declare that the research was conducted in the absence of any commercial or financial relationships that could be construed as a potential conflict of interest.

## Publisher’s Note

All claims expressed in this article are solely those of the authors and do not necessarily represent those of their affiliated organizations, or those of the publisher, the editors and the reviewers. Any product that may be evaluated in this article, or claim that may be made by its manufacturer, is not guaranteed or endorsed by the publisher.

## Abbreviations

CBT: cognitive training
ALE: activation likelihood estimation
DMN: default mode network
ECN: executive control network
SN: salience network
ACC: anterior cingulate
MFG: middle frontal gyrus
DMN: default mode network
ECN: executive control network
SN: salience network
MDD: major depressive disorder
AD: anxiety disorder
OCD: obsessive-compulsive disorder
FAR: Facial Affect Recognition
MRI: magnetic resonance imaging
fMRI: functional magnetic resonance imaging
PET: positron emission tomography
PCC: posterior cingulate cortex
OFC/VLPFC: orbitofrontal cortex/dorsomedial prefrontal cortex
PTSD: post-traumatic stress disorder.
